# Usefulness of Serum Biomarkers in Predicting Anastomotic Leakage After Gastrectomy

**DOI:** 10.3390/cancers17010125

**Published:** 2025-01-03

**Authors:** Diego Ramos, Enrique Gallego-Colón, Javier Mínguez, Ignacio Bodega, Pablo Priego, Francisca García-Moreno

**Affiliations:** 1 General and Gastrointestinal Surgery Department, Hospital Central de la Defensa “Gómez Ulla” CSVE, 28047 Madrid, Spain; ibodega@icloud.com; 2 Department of Medicine, Escuela Militar de Sanidad, 28047 Madrid, Spain; enrique.gce@gmail.com; 3 Hospital Universitario de Getafe, 28905 Getafe, Spain; 4 General and Gastrointestinal Surgery Department, Hospital Universitario Príncipe de Asturias, 28805 Alcalá de Henares, Spain; javyminguez@gmail.com; 5 General and Gastrointestinal Surgery Department, Hospital Universitario La Paz, 28046 Madrid, Spain; papriego@hotmail.com; 6 Biomedical Research Group on Biomaterials and Wound Healing (Ciber-BBN), Traslational Research and Innovation in General and Digestive Surgery (Idipaz), 28046 Madrid, Spain; francisca.garciamoreno@salud.madrid.org

**Keywords:** gastrectomy, anastomotic leakage, biomarker, C-reactive protein, procalcitonin, neutrophil-to-lymphocyte ratio, platelet-to-lymphocyte ratio, fibrinogen, mean platelet volume

## Abstract

Background/Objectives: Anastomotic leakage (AL) is one of the most concerning complications following gastrectomy. The aim of this study was to assess and compare the predictive accuracy of C-reactive protein (CRP), procalcitonin (PCT), the neutrophil-to-lymphocyte ratio (NLR), the platelet-to-lymphocyte ratio (PLR), fibrinogen, and the mean platelet volume (MPV) in the early diagnosis of post-gastrectomy AL. Methods: A prospective bicentric observational study was conducted including all patients undergoing elective gastrectomy between August 2018 and December 2022. The performance of the selected biomarkers in predicting the existence of AL within the first 7 postoperative days (PODs) was assessed. Results: A total of 107 patients were included for analysis. The incidence of AL was 20.56%, and the median day of diagnosis was on POD5 (interquartile range 4–6). CRP, PCT, the NLR, the PLR, and fibrinogen showed significant associations with the presence of AL (from POD2 for CRP and fibrinogen and from POD3 for PCT, NLR, and PLR). CRP demonstrated a superior predictive accuracy on POD4, with a threshold value of 181.4 mg/L (NPV 99%; AUC 0.87, *p* < 0.001); PCT demonstrated a superior predictive accuracy on POD7, with a threshold value of 0.13 μg/L (NPV 98%; AUC 0.84, *p* < 0.001); the NLR showed a superior predictive accuracy on POD6, with a threshold ratio of 6.77 (NPV 95%; AUC 0.86, *p* < 0.001); the PLR achieved a superior predictive accuracy on POD7, with a ratio of 234 (NPV 98%; AUC 0.71; *p* = 0.002); and fibrinogen demonstrated a superior predictive accuracy on POD5, with a threshold of 7.344 g/L (NPV 98%; AUC 0.74; *p* = 0.003). In the comparison of predictive accuracy, CPR, PCT, and the NLR were found to be superior to all other biomarkers. Conclusions: CRP, PCT, and the NLR are biomarkers with a sufficient predictive ability to clinically discard the presence of AL within the first postoperative week.

## Simple Summary

Anastomotic leakage after gastrectomy is a relatively common, but potentially lethal complication, whose morbi-mortality is greatly decreased with a prompt diagnosis and treatment. The aim of this study was to assess the performance of several serum biomarkers in reliably predicting the existence of anastomotic leakage. We confirmed the discrimination capability of C-reactive protein, procalcitonin, the neutrophil-to-lymphocyte ratio, the platelet-to-lymphocyte ratio, and fibrinogen in identifying anastomotic leakage within the first postoperative week. The use of adequate biomarkers has significant implications for optimizing clinical management strategies in these patients and may facilitate the development of future enhanced-recovery programs.

## 1. Introduction

Anastomotic leakage (AL) is one of the most concerning and common complications after gastric surgery, with reported incidence rates ranging from 5 to 20%, or even higher in some series [1,2]. The morbidity and mortality rates of AL are reported to reach 50% [2], and survivors have prolonged hospitalization, increased recurrence rates, and worse long-term functional and oncological outcomes [3,4,5]. The median time of leakage occurrence ranges from 5 to 7 days after gastrectomy [6,7], and an early diagnosis of this complication is crucial for prompt (and, in many cases, aggressive) treatment, with a clear impact on survival and subsequent associated complications [8,9,10]. Many biomarkers have been proposed as potentially valuable tools in the postoperative management of patients undergoing abdominal surgery, including C-reactive protein (CRP), procalcitonin (PCT), a wide variety of cytokines, and many peripheral-blood-cell parameters and indices (such as the neutrophil-to-lymphocyte ratio or platelet indices). Nevertheless, limited data are available on gastric surgery, particularly regarding viable predictors of post-gastrectomy AL, with the available evidence remaining overwhelmingly scarce [11].

Furthermore, newly implemented early-discharge protocols require an adequate detection of patients with postoperative complications prior to discharge, particularly severe or potentially life-threatening AL. Consequently, the development of a post-surgical screening strategy is needed.

Based on these premises, the aim of this study was to determine the roles and predictive accuracies of CRP, PCT, the neutrophil-to-lymphocyte ratio (NLR), the platelet-to-lymphocyte ratio (PLR), the mean platelet volume (MPV), and fibrinogen as early predictors of AL after gastrectomy.

## 2. Materials and Methods

### 2.1. Study Design

A prospective, observational, bicentric cohort study was designed. The participating centers were Ramón y Cajal University Hospital and Príncipe de Asturias University Hospital. This study was reviewed and approved by the Institutional Review Board for both participating centers (approval code: 164-18). Consecutive patients who underwent elective gastrectomy between August 2018 and December 2022 were included in this study. The inclusion criterion was total or near-total (95%) gastrectomy with Roux-en-Y reconstruction, subtotal gastrectomy with Roux-en-Y or Billroth II reconstruction, or proximal gastrectomy followed by esophagogastrostomy. Two abdominal drains were routinely placed for total, near-total, and proximal gastrectomy, and one abdominal drain was routinely placed for subtotal gastrectomy. For patients undergoing total, near-total, or proximal gastrectomy, a routine diatrizoate meglumine (Gastrografin^®^) upper-gastrointestinal study was performed between the 5th and 7th postoperative days (PODs).

The exclusion criteria were an age less than 18 years, emergency surgery, patients undergoing gastric surgery without anastomosis (e.g., wedge resections), bariatric surgery, patients undergoing concomitant resection of other organs, patients with ongoing infection, systemic inflammation or active neoplasms (other than localized and resectable gastric tumors) at the time of surgery, acquired or congenital immunodeficiencies, liver or kidney failure, and inability or refusal to give informed consent.

In all cases, complete blood counts were obtained on a daily basis from POD 1 to 7. Serum CRP levels were measured using immunoturbidimetry (autoanalyzer Alinity^®^-c, Abbott Laboratories, Abbott Park, IL, USA), PCT levels using a chemiluminescence microparticle immunoassay (autoanalyzer Alinity^®^-i, Abbott Laboratories, Abbott Park, IL, USA), and fibrinogen concentration using a colorimetric method (autoanalyzer BCS^®^ XP System, Siemens Healthineers, Erlangen, Germany); and the MPV, NLR, and PLR were determined with an autoanalyzer CELL-DYN^®^ Sapphire (Abbott Laboratories, Abbott Park, IL, USA). The NLR was calculated as the absolute neutrophil count divided by the absolute lymphocyte count. PLR was calculated as the absolute platelet count divided by the absolute lymphocyte count.

### 2.2. Data Collection

For all the patients included in this study, the following data were prospectively collected: age, gender, comorbidities, ASA score, underlying gastric disease, type of operation, surgical approach, operating time, post-gastrectomy AL, other postoperative complications during hospitalization classified according to the Clavien–Dindo score [12], and length of postoperative in-hospital stay.

Diagnostic criteria for AL were defined by changes in drainage fluid (color, turbidity, or enteric/fecal fluid), changes in imaging techniques, or direct visualization of the leak with endoscopy or during reintervention due to peritoneal irritation of patient instability.

CPR, NLR, PLR, fibrinogen, and MPV values were recorded for every POD within the first postoperative week; meanwhile, PCT levels were registered every 48 h (PODs 1, 3, 5, and 7).

### 2.3. Endpoints

The primary endpoint of this study was the assessment of the discriminative and predictive accuracy achieved by CRP, PCT, the NLR, the PLR, fibrinogen, and the MPV within the first postoperative week in determining the occurrence of AL following gastrectomy. The secondary endpoint was to compare the predictive values of all these previously listed variables, trying to establish a gold-standard biomarker for detecting the development of AL.

### 2.4. Data Analysis

Statistical analysis was performed using Stata v.16 for Windows (StataCorp, 2019. Stata Statistical Software: Release 16. StataCorp LLC: College Station, TX, USA). All data were recorded as absolute values and percentages, means and standard deviations (SDs), and median and interquartile range (IQRs), as appropriate, conforming to their category.

Univariate analyses were performed using Student’s *t*-test or the Mann–Whitney *U*-test for quantitative variables, and the c^2^ test or Fisher’s exact test for categorical variables as appropriate. The statistical study was completed with a multivariate analysis using binary logistic regression.

Linear mixed models were used for each of the biomarkers to assess the evolution of the parameters throughout the first postoperative week, using as independent variables AL and POD.

Discrimination was appraised with the area under the curve (AUC) by receiver operating characteristic curves (ROCs). Optimal cut-off points (OCPs) of CRP, PCT, the NLR, the PLR, fibrinogen, and the MPV were calculated by Youden’s J statistics and utilized to determine the sensitivity (Se), specificity (Sp), positive predictive value (PPV), and negative predictive value (NPV). The confidence interval and *p*-value were calculated with DeLong’s method, which was also used to compare AUCs between curves.

In all cases, two-sided *p*-values of <0.05 were considered to be statistically significant.

## 3. Results

### 3.1. Cohort Characteristics

A total of 107 patients undergoing gastrectomy within the study period were recruited. The clinicopathologic features of the enrolled patients are presented in Table 1. There were 50 males (46.73%) and 57 females (53.27%), with a mean age of 73 years (range 64–79). A significant number of patients showed comorbidities, reflected in a high percentage of cases graded ASA II and ASA III according to the American Society of Anesthesiologists physical status classification system (38.32 and 52.34%, respectively) [13]. The vast majority of the patients (89.72%) presented with a diagnosis of gastric adenocarcinoma, of whom 38.32% received neoadjuvant treatment prior to surgery. Overall, 57 patients underwent total or near-total gastrectomy (53.27%), and in most cases, the laparoscopic approach was indicated (67.29%).

The rate of AL was 20.56%, and the median day of AL diagnosis was POD5 (range 4–6). In total, 59.09% of the cases presented as clinical leaks, while the remaining 40.91% were detected through radiological techniques. The reintervention rate was 36.36%, notably higher in the subgroup of patients that presented as clinical leaks (62.50%).

### 3.2. Dynamics of the Inflammatory Markers

**Figure 1:**
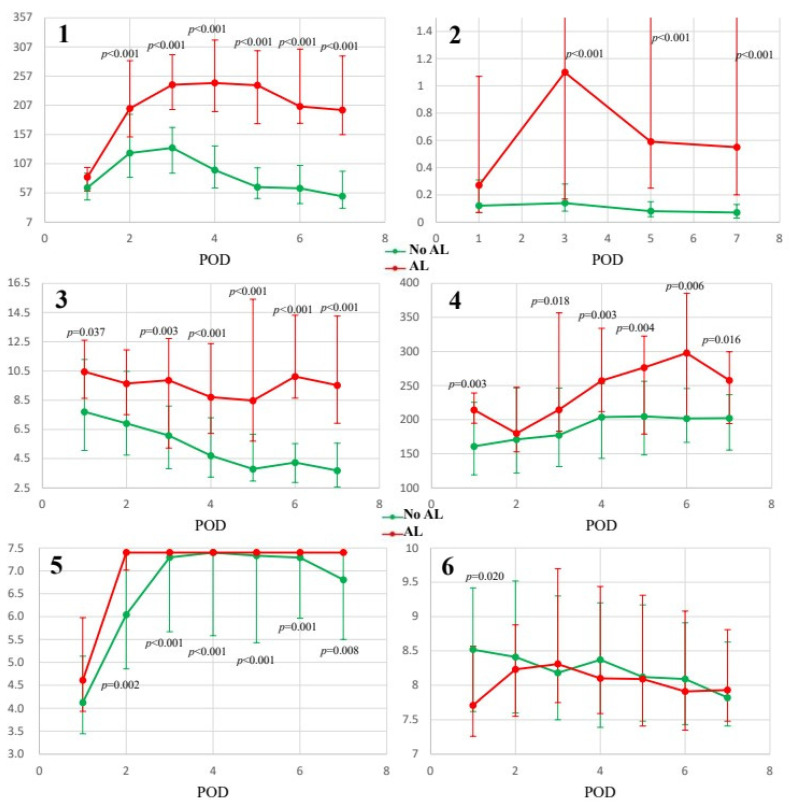
Postoperative changes in inflammatory markers. (**1**): CRP: C-reactive protein (mg/L); (**2**): PCT: procalcitonin (μg/L); (**3**): NLR: neutrophil-to-lymphocyte ratio; (**4**): PLR: platelet-to-lymphocyte ratio; (**5**): fibrinogen (g/L); (**6**): MPV: mean platelet volume (fL). *p*-values were calculated using the Mann–Whitney *U*-test.

The overall levels of the investigated inflammatory biomarkers are shown in Table 2. The CRP levels increased in the first postoperative week, reaching a maximum on POD3, and were significantly higher in the AL group from POD2 to POD7 (*p* < 0.001). The PCT levels did not show any remarkable fluctuation but were found to be significantly higher in the AL group from POD3 to POD7 (*p* < 0.001). The NLR presented a downward trend, significant by POD3, and values in the AL group remained elevated, displaying statistically significant differences from POD3 to POD7 (*p* = 0.003 and *p* < 0.001). The PLR values increased in the AL group from POD3 to POD7 (*p* = 0.018, 0.003, 0.004, 0.006, and 0.016, respectively). The fibrinogen levels showed a significant increase, with maximum levels achieved by POD4, significantly higher in the AL group from POD2 to POD7 (*p* = 0.002, <0.001, and 0.008, respectively). Finally, the MPV levels decreased over time, with statistically significant differences observed only on POD1 (*p* = 0.02) (Figure 1, Table 2 and Table 3).

### 3.3. Predictive Accuracy and Cutoffs

For the detection of AL, a significant discrimination was identified in the ROC curve analysis as early as POD1 for CRP (AUC 0.63; 95%CI 0.5–0.75; cut-off 82.1 mg/L; *p* = 0.037), with best discrimination achieved on POD4 (AUC 0.87; 95%CI 0.77–0.95; cut-off 181.4 mg/L; Se 95%; Sp 90%; PPV 69%; NPV 99%; *p* < 0.001) (Supplemental Table S1). For PCT, significant discrimination was also detected from POD1 (AUC 0.63; 95%CI 0.5–0.75; cut-off 0.22 μg/L; *p* = 0.041), with best discrimination achieved on POD7 (AUC 0.84; 95%CI 0.74–0.91; cut-off 0.1 μg/L; Se 90%; Sp 78%; PPV 56%; NPV 96%; *p* < 0.001) (Supplemental Table S2). Regarding the NLR, significant discrimination was found from POD1 (AUC 0.67; 95%CI 0.57–0.77; cut-off 8.51; *p* = 0.007), with best discrimination identified on POD6 (AUC 0.86; 95%CI 0.76–0.94; cut-off 6.77; Se 86%; Sp 84%; PPV 66%; NPV 95%; *p* < 0.001) (Supplemental Table S3). Concerning the PLR, significant discrimination was also identified from POD1 (AUC 0.73; 95%CI 0.62–0.82; cut-off 190.7; *p* = 0.001), showing best discrimination on POD7 (AUC 0.71; 95%CI 0.58–0.82; cut-off 234; Se 93%; Sp 73%; PPV 47%; NPV 98%; *p* = 0.002) (Supplemental Table S4). For fibrinogen, significant discrimination was observed from POD2 (AUC 0.72; 95%CI 0.61–0.82; cut-off 6.966 g/L; *p* = 0.001), with best discrimination achieved on POD5 (AUC 0.74; 95%CI 0.66–0.8; cut-off 7.344 g/L; Se 95%; Sp 52%; PPV 35%; NPV 98%; *p* = 0.003) (Supplemental Table S5). Finally, no significant discrimination was observed for the MPV on any POD within the first postoperative week.

### 3.4. Comparison of Predictive Accuracy

In the comparison of predictive accuracy among the biomarkers in the prediction of AL, CRP was found to be superior to the PLR on POD2 (*p* = 0.026), to PCT, the NLR, the PLR, and fibrinogen on POD3 (*p* = 0.031, 0.007, 0.005, and <0.001, respectively), and to the NLR, the PLR, and fibrinogen on POD4 (*p* < 0.001); on POD5, CRP showed better accuracy than the NLR, the PLR, and fibrinogen (*p* = 0.008, 0.002, and <0.001, respectively), and PCT was found to be superior to the PLR and fibrinogen (*p* = 0.039 and 0.026, respectively); on POD6, both CRP and the NLR showed a higher accuracy than the PLR and fibrinogen (*p* < 0.001); and on POD7, CRP, PCT, and the NLR were found to be more accurate than the PLR and fibrinogen (*p* < 0.001, 0.011, 0.025, 0.003, and 0.007, respectively).

## 4. Discussion

In this study, we analyzed and compared the predictive accuracies of multiple biomarkers including CRP, PCT, the NLR, the PLR, fibrinogen, and the MPV in determining the occurrence of AL following gastrectomy in a prospective study. According to our results, all these biomarkers, with the exception of the MPV, showed significant predictive accuracy for the detection of AL in the first postoperative week, with the most reliable markers CRP, PCT, and the NLR. As far as the PLR and fibrinogen are concerned, although statistical significance was achieved, their clinical relevance remains to be validated, given their lower performance and relatively low AUCs until later PODs.

In our study, CRP achieved the higher discrimination on POD4; however, the median day of AL diagnosis was POD5, which aligns with previously published evidence. These studies have demonstrated that, as early as POD3, the inflammatory response to resection is attenuated in patients with a normal postoperative course. Consequently, elevated CRP could indicate the presence of a postoperative infectious complication [8,11,14]. Based on these findings, we propose that a higher clinical discriminatory threshold could be achieved on POD3.

As an acute phase reactant molecule, CPR has been proposed as a biomarker candidate for numerous conditions. There is substantial evidence of the utility of CRP in detecting complications following different abdominal procedures, essentially related to infectious complications and AL in colorectal surgery [15,16], but with respect to gastric surgery, data are still scarce. There is, however, growing evidence of the utility of CRP to detect patients with a low risk of infectious intraabdominal complications [7,8,9,17], yet only limited studies have evaluated the predictive accuracy of CRP to identify AL occurrence. These studies support the potential utility of CRP in AL diagnosis following gastrectomy; however, there is ongoing controversy regarding the optimal POD for AL diagnosis and the corresponding discriminatory thresholds, which range from POD to POD7, and serum levels from 94 to 209 mg/L [7,18,19,20,21]. Our study provides evidence of a potential use of CRP as a biomarker following gastric surgery, and we propose CRP as a clinically relevant biomarker for AL diagnosis, with an identified threshold of 162.4 mg/L.

PCT showed the higher predictive accuracy by POD7, although higher clinical discrimination was observed by POD3, and thus a predictive threshold value of 0.4 μg/L is advised. PCT recently showed promising results in detecting intra-abdominal complications following esophagogastric surgery [22,23,24] but, to date, only Cananzi et al. [7] have specifically investigated PCT in relation to AL diagnosis in gastric surgery, reporting similar results to our study and identifying significant discrimination from POD6, a noticeably later date than ours, and with the best discrimination achieved on POD7 (AUC 0.763, 95%CI 0.684–0.831, cut-off 0.4 μg/L, NPV 97%, *p* = 0.002).

The NLR demonstrated superior discrimination in our study on POD6; however, we propose POD3 for a higher clinical relevance. Most of the published evidence on the NLR is focused on its quantification in the preoperative period [25,26], rather than assessing the predictive accuracy in the postoperative period, and only two series have studied the implications of postoperative fluctuations. In a retrospective study, Clemente-Gutierrez et al. identified that the NLR predicted the occurrence of AL on POD3 (AUC 0.78, cut-off 10, NPV 96%), but only by defining patients who underwent total gastrectomy with esophagojejunostomy and required invasive management as AL [27]. Similarly, another retrospective series of Çetin et al. [28] described that the NLR was significantly higher in patients with AL and other postoperative complications (*p* = 0.022), although its predictive accuracy was not assessed. Consequently, we suggest that the NLR levels on POD3 could represent an interesting biomarker for AL following gastric surgery, with a suggested threshold of 8.86.

This is the first prospective study to evaluate the usefulness of CRP, PCT, the NLR, the PLR, fibrinogen, and the MPV in the early postoperative period after gastrectomy, and to compare their accuracy, and our results have marked implications for clinical practice. When comparing the predictive accuracies on POD3, CRP was found to be superior to PCT and the NLR, so it could be proposed as the biomarker of choice to determine the occurrence of AL on that POD. This contrasts with previous evidence in colorectal surgery, in which PCT proved to be more accurate than CRP in predicting AL [29]. The most feasible explanation is the substantial difference in gut microbiota between the proximal and distal segments of the gastrointestinal tract, as observed in bacterial gut growth cultures, abscesses, and the bloodstream after abdominal surgical complications. Hence, after gastric surgery, *Candida* spp., *Klebsiella pneumoniae*, *Streptococcus*, and *Staphylococcus* spp. are often isolated, in contrast to a strikingly low rate of fungal infections and a prominent role of Gram-negative bacteria following colorectal surgery [7], with PCT specifically triggered by the latter and dramatically less by Gram-positive bacteria or fungi [30].

A direct recommendation we can make based on the foregoing is that PCT should not be routinely determined during the normal postoperative course following gastric resection to discard AL, both considering the lower accuracy when compared with CRP and the elevated economic cost, ranging from four to seven times higher than that of CRP [31,32], which combined raise concerns regarding its cost-effectiveness balance.

Additionally, the NLR proved to be less accurate than CRP, as well as PCT (but not inferior to the latter). However, contrary to PCT determination, a complete blood count is an inexpensive test routinely performed throughout the postoperative period. As such, the NLR could be a useful biomarker adjuvant to the interpretation of CRP values.

Finally, regardless of the proposed threshold on POD3 for improved clinical discrimination, CRP was identified as a valuable negative predictive biomarker for AL from POD2 to POD7, showing remarkable potential to identify low-risk patients in the recovery process [21]. Consequently, CRP monitoring could substantially enhance the risk stratification of patients, enabling the identification of low-risk patients, eligible for fast-tracking and early discharge. Furthermore, combining CRP with the NLR could facilitated the development of a composite risk score, potentially improving stratification compared to CRP alone. This is particularly relevant in the field of gastric surgery, where prolonged hospitalization is common and enhanced recovery after surgery (ERAS) protocols have not been widely implemented, mainly due to concerns about patient safety and unclear benefits in terms of readmission rates [7,33].

It is, indeed, worth mentioning that any reintervention, regardless of the cause, involves a new surgical insult, leading to an elevation of inflammatory markers. However, reinterventions are consistently performed after a diagnosis of AL, meaning that any increase in the relevant biomarkers that occurs subsequently has an extremely low likelihood of affecting their early predictive accuracy.

This study has several limitations, including the selection of biomarkers analyzed, which may have excluded other clinically relevant markers, as well as the absence of external validation for the results. The population of this study also showed a relatively high rate of AL, though negative predictive values actually increase as the prevalence of the tested event decreases [34]; this fact should not affect the results, but rather support them. Baseline preoperative levels of the biomarkers were not measured, limiting our understanding of the dynamic changes that occur and may be used to diagnose AL. Notwithstanding its limitations, this is to our knowledge the first prospective study to simultaneously evaluate various inflammatory biomarkers, such as CRP, PCT, the NLR, the PLR, fibrinogen, and the MPV, and compare their predictive accuracy to determine the occurrence of AL following gastric resection. The results are valuable, as some biomarkers were decisively discarded, while CRP emerged as the biomarker of choice for clinical application, providing a foundation for the development of future combined risk scores.

## 5. Conclusions

CPR, PCT, and the NLR demonstrated significant discrimination and predictive accuracy in determining the occurrence of AL following gastrectomy within the first postoperative week.

CRP had a better performance than PCT and the NLR and thus should be used as the reference screening postoperative biomarker in the studied population. CRP-based protocols could be further developed to optimize postoperative management.

## Figures and Tables

**Table 1:** Clinicopathologic characteristics of the enrolled patients.

		Overall (*n* = 107)	Patients Without AL (*n* = 85)	Patients with AL (*n* = 22)	*p*-Value
Age (years)		73 (64–79)	73 (64–78)	74 (68–80)	0.20
Gender					
	Male	50 (46.73%)	37 (43.53%)	13 (59.09%)	0.19
	Female	57 (53.27%)	48 (56.47%)	9 (40.91%)	
ASA score					
	I	6 (5.61%)	6 (7.06%)	0 (0.00%)	0.64
	II	46 (38.32%)	32 (37.65%)	9 (40.91%)	
	III	36 (52.34%)	44 (51.76%)	12 (54.55%)	
	IV	4 (6.54%)	3 (3.53%)	1 (4.55%)	
Histology					
	ADC	96 (89.72%)	76 (89.41%)	30 (90.91%)	0.83
	GIST	3 (2.80%)	2 (2.35%)	1 (4.55%)	
	PUD	2 (1.87%)	2 (2.35%)	0 (0.00%)	
	Other	6 (5.61%)	5 (5.88%)	1 (4.55%)	
NAT		41 (38.32%)	37 (43.53%)	4 (18.18%)	0.029
Type of gastrectomy					
	Total	44 (41.12%)	31 (36.47%)	13 (59.09%)	0.28
	Near-total (95%)	13 (12.15%)	11 (12.94%)	2 (9.09%)	
	Subtotal	49 (45.79%)	42 (49.41%)	7 (31.82%)	
	Proximal	1 (0.93%)	1 (1.18%)	0 (0.00%)	
Surgical approach					
	Open	28 (26.17%)	22 (25.88%)	6 (27.27%)	0.002
	Laparoscopic	72 (67.29%)	61 (71.76%)	11 (50.00%)	
	LCO	7 (6.54%)	2 (2.35%)	5 (22.73%)	
Procedure duration (min)		290 (246–334)	280 (240–324)	320 (263–370)	0.010
Other complications		66 (61.68%)	49 (57.65%)	17 (77.27%)	0.091
Clavien–Dindo score					
	I	19 (26.03%)	19 (22.35%)	0 (0.00%)	<0.001
	II	33 (45.21%)	26 (30.59%)	7 (31.82%)	
	III	8 (10.96%)	2 (2.35%)	6 (27.27%)	
	IV	4 (5.48%)	2 (2.35%)	2 (9.09%)	
	V	9 (12.33%)	2 (2.35%)	7 (31.82%)	
Postoperative stay (days)		10 (7–17)	9 (7–12)	25 (17–39)	<0.001
Mortality		9 (8.41%)	2 (2.35%)	7 (31.82%)	<0.001

AL: anastomotic leakage; ASA: American Society of Anesthesiologists; ADC: gastric adenocarcinoma; GIST: gastrointestinal stromal tumor; PUD: peptic ulcer disease; NAT: neoadjuvant treatment; LCO: laparoscopy with conversion to open surgery.

**Table 2:** Dynamics of the inflammatory biomarkers.

	CRP			PCT			NLR		
	Coefficient	95% CI	*p*	Coefficient	95% CI	*p*	Coefficient	95% CI	*p*
POD1	BL	BL	NA	BL	BL	NA	BL	BL	NA
POD2	77.60	63.34–91.87	<0.001	NA	NA	NA	0.49	(−)1.46–1.56	0.949
POD3	86.94	72.72–101.17	<0.001	0.22	(−)1.70–2.15	0.821	−1.61	(−)3.11–(−)0.10	0.036
POD4	61.65	47.31–74.99	<0.001	NA	NA	NA	−2.82	(−)4.34–(−)1.30	<0.001
POD5	37.38	22.92–51.85	<0.001	1.45	(−)0.49–3.39	0.145	−3.52	(−)5.04–(−)1.99	<0.001
POD6	31.44	16.13–46.75	<0.001	NA	NA	NA	−3.41	(−)5.02–(−)1.79	<0.001
POD7	21.21	6.68–36.73	0.007	0.46	(−)1.61–2.53	0.663	−3.31	(−)4.96–(−)1.67	<0.001
AL	115.37	92.87–137.88	<0.001	3.43	1.72–5.15	<0.001	4.27	2.43–6.12	<0.001
	**PLR**			**Fibrinogen**			**MPV**		
	**Coefficient**	**95% CI**	***p***	**Coefficient**	**95% CI**	***p***	**Coefficient**	**95% CI**	***p***
POD1	BL	BL	NA	BL	BL	NA	BL	BL	NA
POD2	19.16	(−)17.43–55.75	0.305	167.04	145.956–188.12	<0.001	−0.66	(−)1.44–0.13	0.101
POD3	8.65	(−)27.84–45.14	0.642	219.77	198.62–240.91	<0.001	−0.62	(−)1.40–0.16	0.120
POD4	16.74	(−)20.05–53.53	0.372	222.94	201.73–244.15	<0.001	−0.86	(−)1.65–(-)0.07	0.033
POD5	14.15	(−)22.85–51.14	0.454	215.92	194.57–237.27	<0.001	−0.85	(−)1.66–(-)0.06	0.035
POD6	24.12	(−)15.11–63.34	0.228	214.39	191.82–236.97	<0.001	−0.88	(−)1.72–(−)0.04	0.040
POD7	1.91	(−)37.92–41.73	0.925	181.87	158.96–204.77	<0.001	−0.97	(−)1.82-(−)0.12	0.026
AL	60.49	12.07–108.91	0.014	80.94	35.12–126.76	0.001	−0.35	(−)1.17–(+)0.46	0.398

Linear mixed-model study for each individual biomarker using AL (anastomotic leakage) and POD (postoperative day) as independent variables. CRP: C-reactive protein; PCT: procalcitonin; NLR: neutrophil-to-lymphocyte ratio; PLR: platelet-to-lymphocyte ratio; MPV: mean platelet volume; BL: baseline; NA: not available.

**Table 3:** Binary logistic regression of inflammatory biomarker levels.

	CRP		PCT		NLR	
	OR (95% CI)	*p*	OR (95% CI)	*p*	OR (95% CI)	*p*
POD1	NA	NA	NA	NA	1.039 (0.974–1.109)	0.244
POD2	1.011 (1.005–1.017)	<0.001	NA	NA	NA	NA
POD3	1.018 (1.010–1.026)	<0.001	1.451 (1.030–2.043)	0.033	1.076 (1.000–1.157)	0.05
POD4	1.025 (1.015–1.035)	<0.001	NA	NA	1.161 (1.049–1.285)	0.004
POD5	1.025 (1.015–1.035)	<0.001	6.831 (1.865–25.023)	0.004	1.333 (1.160–1.533)	<0.001
POD6	1.032 (1.018–1.047)	<0.001	NA	NA	1.681 (1.326–2.130)	<0.001
POD7	1.030 (1.016–1.045)	<0.001	18.83 (2.45–144.51)	0.005	1.585 (1.259–1.994)	<0.001
	**PLR**		**Fibrinogen**		**MPV**	
	**OR (95% CI)**	***p***	**OR (95% CI)**	***p***	**OR (95% CI)**	***p***
POD1	1.002 (0.999–1.005)	0.229	NA	NA	0.589 (0.384–0.904)	0.015
POD2	NA	NA	1.007 (1.002–1.012)	0.006	NA	NA
POD3	1.005 (1.001–1.009)	0.008	1.015 (1.003–1.028)	0.018	NA	NA
POD4	1.007 (1.002–1.012)	0.007	1.020 (1.001–1.039)	0.036	NA	NA
POD5	1.007 (1.002–1.012)	0.006	1.019 (1.002–1.035)	0.027	NA	NA
POD6	1.006 (1.001–1.010)	0.014	1.013 (1.001–1.025)	0.029	NA	NA
POD7	1.005 (0.999–1.010)	0.06	1.002 (0.998–1.006)	0.286	NA	NA

Only values that previously showed statistical significance in the Mann-Whitney *U*-test were analyzed. CRP: C-reactive protein; PCT: procalcitonin; NLR: neutrophil-to-lymphocyte ratio; PLR: platelet-to-lymphocyte ratio; MPV: mean platelet volume; POD: postoperative day.

## Data Availability

The original contributions presented in this study are included in the article/Supplementary Material. Further inquiries can be directed to the corresponding author.

## Supplementary Materials

The following supporting information can be downloaded at https://www.mdpi.com/article/10.3390/cancers17010125/s1, Table S1: ROC curve analysis for CRP levels; Table S2: ROC curve analysis for PCT levels; Table S3: ROC curve analysis for NLR values; Table S4: ROC curve analysis for PLR values; Table S5: ROC curve analysis for fibrinogen levels.
