# Supplementary material for: Usefulness of Serum Biomarkers in Predicting Anastomotic Leakage After Gastrectomy
Source: Cancers (Basel). 2025 Jan 3;17(1):125. doi: 10.3390/cancers17010125 (PMC11720279; doi:10.3390/cancers17010125)
Supplement: Supplementary file 1 [file cancers-17-00125-s001.zip › cancers-3363352-supplementary.pdf]

	n	Prevalence	Cut-off	Se (95% CI)	Sp (95% CI)	PPV (95% CI)	NPV (95% CI)	AUC (95% CI)	Youden Index (95% CI)	p_{AUC}
**POD1**	101	19.8%	82.1mg/L	0.55 (0.31 - 0.79)	0.7 (0.59 - 0.81)	0.31 (0.15 - 0.48)	0.86 (0.77 - 0.95)	0.63 (0.5 - 0.75)	0.25 (0.01 - 0.49)	**0.037**
**POD2**	105	20.9%	144.5 mg/L	0.82 (0.63 - 1)	0.62 (0.51 - 0.73)	0.36 (0.22 - 0.5)	0.93 (0.85 - 1)	0.72 (0.62 - 0.81)	0.43 (0.24 – 0.62)	**0.001**
**POD3**	106	20.8%	162.4 mg/L	0.91 (0.77 - 1)	0.71 (0.61 - 0.82)	0.46 (0.29 - 0.61)	0.97 (0.92 - 1)	0.82 (0.73 - 0.89)	0.62 (0.47 – 0.78)	**<0.001**
**POD4**	103	19%	181.4 mg/L	0.95 (0.82 - 1)	0.9 (0.83 - 0.97)	0.69 (0.49 - 0.89)	0.99 (0.95 - 1)	0.87 (0.77 - 0.95)	0.85 (0.73 – 0.97)	**<0.001**
**POD5**	99	21.2%	151.3 mg/L	0.81 (0.62 - 1)	0.91 (0.84 - 0.98)	0.71 (0.51 - 0.91)	0.95 (0.89 - 1)	0.86 (0.77 - 0.95)	0.72 (0.54 – 0.9)	**<0.001**
**POD6**	83	26.5%	159.2 mg/L	0.91 (0.77 - 1)	0.89 (0.79 - 0.97)	0.74 (0.56 - 0.93)	0.96 (0.91 - 1)	0.91 (0.82 - 0.97)	0.79 (0.65 – 0.94)	**<0.001**
**POD7**	79	26.6%	112.3 mg/L	0.91 (0.76 - 0.92)	0.81 (0.7 - 0.92)	0.63 (0.44 - 0.82)	0.96 (0.89 - 1)	0.87 (0.78 - 0.94)	0.72 (0.55 – 0.88)	**<0.001**

Supplemental Table S1: ROC curve analysis for CRP levels. Se: sensitivity; Sp: specificity; PPV: positive predictive value; NPV: negative predictive value; AUC: area under the curve; POD: postoperative day.

	n	Prevalence	Cut-off	Se (95% CI)	Sp (95% CI)	PPV (95% CI)	NPV (95% CI)	AUC (95% CI)	Youden Index (95% CI)	p_{AUC}
**POD1**	103	19.4%	0.22 µg/L	0.55 (0.31 – 0.79)	0.71 (0.59 – 0.8)	0.31 (0.14 – 0.47)	0.87 (0.78 – 0.95)	0.63 (0.5 – 0.75)	0.25 (0.01 – 0.49)	**0.041**
**POD3**	105	20%	0.4 µg/L	0.67 (0.44 – 0.89)	0.81 (0.71 – 0.89)	0.45 (0.26 – 0.64)	0.91 (0.83 – 0.98)	0.74 (0.62 – 0.84)	0.46 (0.25 – 0.68)	**<0.001**
**POD5**	101	21.8%	0.17 µg/L	0.82 (0.63 – 1)	0.82 (0.7 – 0.89)	0.53 (0.35 – 0.71)	0.94 (0.88 – 1)	0.82 (0.71 – 0.9)	0.62 (0.43 – 0.8)	**<0.001**
**POD7**	80	26.3%	0.13 µg/L	0.9 (0.76 – 1)	0.78 (0.62 – 0.86)	0.56 (0.38 – 0.74)	0.96 (0.89 – 1)	0.84 (0.74 – 0.91)	0.65 (0.48 – 0.82)	**<0.001**

Supplemental Table S2: ROC curve analysis for PCT levels. Se: sensitivity; Sp: specificity; PPV: positive predictive value; NPV: negative predictive value; AUC: area under the curve; POD: postoperative day.

	n	Prevalence	Cut-off	Se (95% CI)	Sp (95% CI)	PPV (95% CI)	NPV (95% CI)	AUC (95% CI)	Youden Index (95% CI)	p_{AUC}
**POD1**	107	20.6%	8.51	0.77 (0.58 – 0.97)	0.58 (0.45 – 0.68)	0.32 (0.18 – 0.45)	0.91 (0.82 – 0.99)	0.67 (0.57 – 0.77)	0.34 (0.13 – 0.54)	**0.007**
**POD2**	106	20.8%	8.59	0.59 (0.36 – 0.82)	0.69 (0.59 – 0.8)	0.33 (0.17 – 0.49)	0.87 (0.78 – 0.96)	0.65 (0.52 – 0.76)	0.28 (0.05 – 0.51)	**0.018**
**POD3**	107	20.6%	8.86	0.64 (0.41 – 0.86)	0.8 (0.71 – 0.89)	0.45 (0.26 – 0.64)	0.9 (0.82 – 0.97)	0.72 (0.61 – 0.83)	0.44 (0.22 – 0.65)	**<0.001**
**POD4**	104	21.2%	7.38	0.73 (0.52 – 0.94)	0.76 (0.66 – 0.86)	0.44 (0.27 – 0.62)	0.91 (0.84 – 0.99)	0.75 (0.64 – 0.85)	0.48 (0.28 – 0.69)	**<0.001**
**POD5**	102	21.6%	5.46	0.82 (0.63 – 1)	0.72 (0.61 – 0.82)	0.44 (0.28 – 0.6)	0.93 (0.86 – 1)	0.77 (0.67 – 0.86)	0.53 (0.34 – 0.72)	**<0.001**
**POD6**	84	26.2%	6.77	0.86 (0.69 – 1)	0.84 (0.74 – 0.94)	0.66 (0.47 – 0.85)	0.95 (0.88 – 1)	0.86 (0.76 – 0.94)	0.7 (0.53 – 0.87)	**<0.001**
**POD7**	80	20.3%	4.63	0.95 (0.84 – 1)	0.71 (0.59 – 0.84)	0.54 (0.37 – 0.72)	0.98 (0.92 – 1)	0.84 (0.76 – 0.91)	0.66 (0.52 – 0.81)	**<0.001**

Supplemental Table S3: ROC curve analysis for NLR values. Se: sensitivity; Sp: specificity; PPV: positive predictive value; NPV: negative predictive value; AUC: area under the curve; POD: postoperative day.

	n	Prevalence	Cut-off	Se (95% CI)	Sp (95% CI)	PPV (95% CI)	NPV (95% CI)	AUC (95% CI)	Youden Index (95% CI)	p_{AUC}
**POD1**	107	20.6%	190.7	0.82 (0.63 – 1)	0.64 (0.52 – 0.73)	0.36 (0.22 – 0.5)	0.93 (0.86 – 1)	0.73 (0.62 – 0.82)	0.44 (0.25 – 0.63)	**0.001**
**POD2**	106	20.8%	144.3	0.91 (0.77 – 1)	0.61 (0.49 – 0.72)	0.38 (0.24 – 0.52)	0.96 (0.9 – 1)	0.66 (0.57 – 0.73)	0.52 (0.36 – 0.68)	**0.016**
**POD3**	107	20.6%	181.93	0.77 (0.58 – 0.97)	0.54 (0.42 – 0.64)	0.3 (0.17 – 0.43)	0.9 (0.81 – 0.99)	0.66 (0.55 – 0.76)	0.3 (0.1 – 0.51)	**0.015**
**POD4**	104	21.2%	230.26	0.64 (0.41 – 0.86)	0.68 (0.56 – 0.78)	0.34 (0.18 – 0.49)	0.87 (0.78 – 0.96)	0.66 (0.54 – 0.77)	0.31 (0.08 – 0.53)	**0.011**
**POD5**	102	21.6%	238.75	0.68 (0.47 – 0.9)	0.68 (0.55 – 0.77)	0.36 (0.2 – 0.51)	0.88 (0.79 – 0.97)	0.68 (0.56 – 0.78)	0.34 (0.12 – 0.56)	**0.005**
**POD6**	84	26.2%	287.06	0.55 (0.32 – 0.78)	0.89 (0.78 – 0.96)	0.6 (0.36 – 0.84)	0.84 (0.75 – 0.94)	0.72 (0.59 – 0.82)	0.42 (0.19 – 0.64)	**<0.001**
**POD7**	80	20.3%	234	0.93 (0.77 – 1)	0.73 (0.61 - 0.85)	0.47 (0.27 – 0.66)	0.98 (0.92 – 1)	0.71 (0.58 – 0.82)	0.66 (0.49 – 0.83)	**0.002**

Supplemental Table S4: ROC curve analysis for PLR values. Se: sensitivity; Sp: specificity; PPV: positive predictive value; NPV: negative predictive value; AUC: area under the curve; POD: postoperative day.

	n	Prevalence	Cut-off	Se (95% CI)	Sp (95% CI)	PPV (95% CI)	NPV (95% CI)	AUC (95% CI)	Youden Index (95% CI)	p_{AUC}
**POD1**	105	20.9%	4.128 g/L	0.68 (0.47 – 0.9)	0.52 (0.39 – 0.62)	0.27 (0.14 – 0.39)	0.86 (0.75 – 0.97)	0.6 (0.48 – 0.71)	0.19 (-0.03 – 0.41)	0.121
**POD2**	105	21%	6.966 g/L	0.77 (0.58 – 0.97)	0.66 (0.54 – 0.76)	0.37 (0.22 – 0.52)	0.92 (0.84 – 0.99)	0.72 (0.61 – 0.82)	0.42 (0.22 – 0.63)	**0.001**
**POD3**	104	20.2%	7.342 g/L	0.91 (0.76 – 1)	0.52 (0.39 – 0.62)	0.32 (0.19 – 0.44)	0.95 (0.88 – 1)	0.71 (0.62 – 0.79)	0.41 (0.25 – 0.58)	**0.003**
**POD4**	103	21.4%	7.28 g/L	0.91 (0.77 – 1)	0.48 (0.35 – 0.58)	0.32 (0.19 – 0.44)	0.95 (0.87 – 1)	0.7 (0.61 – 0.77)	0.38 (0.22 – 0.54)	**0.005**
**POD5**	101	21.8%	7.344 g/L	0.95 (0.85 – 1)	0.52 (0.39 – 0.62)	0.35 (0.22 – 0.48)	0.98 (0.92 – 1)	0.74 (0.66 – 0.8)	0.46 (0.32 – 0.6)	**0.003**
**POD6**	84	26.2%	7.369 g/L	0.86 (0.7 – 1)	0.55 (0.4 – 0.66)	0.4 (0.25 – 0.54)	0.92 (0.81 – 1)	0.71 (0.6 – 0.79)	0.4 (0.21 – 0.59)	**0.003**
**POD7**	80	26.3%	7.163 g/L	0.81 (0.62 – 1)	0.63 (0.48 – 0.74)	0.43 (0.26 – 0.59)	0.9 (0.79 – 1)	0.72 (0.6 – 0.82)	0.42 (0.21 – 0.63)	**0.002**

Supplemental Table S5: ROC curve analysis for fibrinogen levels. Se: sensitivity; Sp: specificity; PPV: positive predictive value; NPV: negative predictive value; AUC: area under the curve; POD: postoperative day.
